# Associations of *nicotidamide-N-methyltransferase*, *FTO*, and *IRX3* genetic variants with body mass index and resting energy expenditure in Mexican subjects

**DOI:** 10.1038/s41598-020-67832-7

**Published:** 2020-07-10

**Authors:** Maciel Bañales-Luna, Nicté Figueroa-Vega, Carolina Ivet Marín-Aragón, Elva Perez-Luque, Lorena Ibarra-Reynoso, Hugo L. Gallardo-Blanco, Itzel López-Aguilar, Juan Manuel Malacara

**Affiliations:** 1 Department of Medical Sciences, Division of Health Sciences, University of Guanajuato, León Campus, 20 de Enero #929, Colonia Obregón, C.P. 37320 León, Guanajuato Mexico; 2 Department of Genetics, School of Medicine, University Autonomous of Nuevo León, Monterrey, N.L. Mexico

**Keywords:** Haplotypes, Obesity

## Abstract

The enzyme nicotidamide-N-methyltransferase (NNMT) regulates adipose tissue energy expenditure through increasing nicotinamide adenosine dinucleotide (NAD^+^) content. NNMT methylates nicotinamide to *N*^*1*^-methylnicotidamide (MNA-1) using S-adenosyl methionine. The *rs*694539 *NNMT* polymorphism is associated with non-alcoholic steatohepatitis, and *rs*1941404 is associated with hyperlipidemia. The *rs*1421085 *FTO* is related to poor eating behaviors, and rs3751723 *IRX3* is associated with obesity. To investigate the association of rs694539 and rs1941404 *NNMT*, rs140285 *FTO* and rs3751723 *IRX3* polymorphisms with MNA-1 concentrations, resting energy expenditure (REE) and BMI, we included clinically healthy Mexican subjects 30 to 50 years old, 100 subjects (35 men/65 women) with BMI > 30 kg/m^2^ and 100 subjects (32 men/68 women) with BMI < 25 kg/m^2^. Glucose, lipid profile, insulin, leptin, acylated ghrelin, and MNA-1 (LC–MS) were quantified. Resting energy expenditure (REE) was estimated using indirect calorimetry with a Fitmate instrument. Genotyping was performed using PCR–RFLP, and allelic discrimination was examined using TaqMan probes. MNA-1 concentrations and REE were significantly higher in obese subjects. Subjects with the rs694539AA *NNMT* genotype (recessive model) had lower weight, BMI, and REE. BMI showed an association with HDL-C, triglycerides, MNA-1, acetylated ghrelin, leptin, insulin concentrations, HOMA-IR, REE, and rs1421085. Subjects with the TC or CC genotypes of rs1421085 *FTO* showed 6 kg and 2 units of BMI more than did those with the TT wild type. The CG of the rs1421085 and rs3751723 haplotypes was associated with BMI. These findings showed that BMI was strongly associated with REE, rs1421085 *FTO* and the CG rs1421085 *FTO* and rs3751723 *IRX3* haplotypes. We used the GMDR approach in obesity phenotype to show the interaction of four SNPs and metabolic variables.

## Introduction

Obesity has become a global epidemic leading to increased cardiovascular disease, type 2 diabetes, cancer, and others associated pathologies^1^. Seven of ten adults are obese or overweight (combined prevalence of 72.5%) in Mexico^2^. A persistent state of positive energy balance when food intake consistently exceeds energy expenditure (REE) leads to weight gain and eventually to obesity onset^3^. Several determinants of 24-h REE, such as body fat-free mass, fat mass, age, gender, ethnicity, glucose tolerance, and familial relationship, reflect an underlying genetic background^4–6^. The nicotidamide-*N*-methyltransferase (NNMT) enzyme is a novel regulator of adiposity and energy expenditure, and it directly alters with NAD^+^ and SAM, which affects histone methylation, polyamine flux and SIRT1 signaling^7^. Levels of the NNMT enzyme are high in adipose tissue and liver and low in other organs^7,8^. *NNMT*-knockdown mice show an increased loss of body fat, as indicated by increased energy expenditure and oxygen consumption^7^. NNMT inhibition by *N*^*1*^-methylnicotidamide, which is the product of the NNMT reaction, increases oxygen consumption in adipocytes^7^. The expression of *NNMT* in white adipose tissue is approximately two-fold higher in type 2 diabetes mellitus patients (T2DM) than it is in healthy controls^9^, with elevated levels of MNA-1 observed in the circulation^9,10^ and urine^11^. The MNA-1 plasma levels correlate positively with BMI^9,11^ and inversely with insulin sensitivity^9^.

The NNMT enzyme is encoded by the *NNMT* gene located on 11q23.1, which harbors several single nucleotide polymorphisms (SNPs). Two recently identified SNPs, rs694539 and rs1941404, were associated with chronic diseases such as hyperhomocysteinemia^12^, nonalcoholic steatohepatitis^13^, congenital heart disease^14^, bipolar disorder^15^, and epilepsy^16^. The rs1941404 was associated significantly with hyperlipidemia. The effect of rs1941404 variations on REE may be a primary physiological mechanism that leads to hyperlipidemia^17^. However, the associations of the rs694539 and rs1941404 variations with obesity and resting energy expenditure were not examined.

Sequence variants in the first intron of *FTO* are strongly associated with human obesity, and carriers of the risk alleles show evidence of increased appetite and food intake^18,19^. A recent investigation showed that the rs1421085 risk allele of the *FTO* gene disrupted ARID5B (AT-rich interactive domain-containing protein 5B) repressor binding, which led to derepression of *IRX3 (*Iroquois homeobox protein 3*)* and *IRX5* expression during early adipocyte differentiation, triggering alterations in browning^20^. The risk alleles of the *FTO* rs1421085 polymorphisms are associated with poor eating behaviors (higher hunger, and emotional disinhibiting scores), a higher intake of high fat foods and refined starches with depressive symptoms^21^. The obesity-associated SNPs in the first intron of *FTO* are functionally connected with the IRX3 gene. These obesity-associated SNPs directly interact with promoters of *FTO* and *IRX3* in humans^22^. *IRX3*-deficient mice have a 25–30% lower body weight, which suggests a role in the increase in basal metabolic rate^23^.

The present study investigated the association of rs694539 and rs1941404 of the *NNMT* gene, rs140285 *FTO* and rs3751723 *IRX3* polymorphisms with serum MNA-1 concentrations, REE, and BMI in lean and obese subjects.

## Results

The anthropometric and metabolic characteristics of the study groups are shown in Table 1. Circulating MNA-1 and REE were significantly higher in obese subjects. The other metabolic variables showed the expected differences between lean and obese subjects. There was a strong association between REE and BMI (R = 0.42, t = 6.6377859, *p* < 0.00001).

**Table 1:** Anthropometric and metabolic characteristics of the study subjects.

	Lean subjects n = 100	Obese subjects n = 100	t/z	*p*
Age (years)	30 ± 7	36 ± 8	-5.1	< 0.00001
Weight (kg)	62 ± 9	91 ± 14	-10.5	< 0.0001
BMI (kg/m^2^)	22.5 ± 2	34 ± 4	-16.0	< 0.0001
SBP (mmHg)	107 ± 10	114 ± 9	-2.6	0.01
DBP (mmHg)	71 ± 8	77 ± 6.5	-3.2	0.001
Glucose (mg/dL)	85 ± 11	91 ± 12	-1.1	NS
Total cholesterol (mg/dL)	175 ± 32	184 ± 36	-0.4	NS
HDL-c (mg/dL)	57 ± 14	46 ± 14	3.9	0.001
LDL-c (mg/dL)	98 ± 29	105 ± 41	-1.02	NS
Triglycerides (mg/dL)	89 (61–123)	138 (95–180)	-2.98	< 0.01
Insulin (µIU/mL)	8.5 (6–10)	15 (10–21)	-4.32	< 0.0001
Acylated ghrelin (pg/mL)	112 (84–160)	76 (37–132)	-2.78	< 0.01
Leptin (ng/mL)	16 (8–30)	47 (27–65)	-3.68	< 0.001
HOMA-IR	1.7 (1.3–2.2)	3.0 (2.3–5)	-4.2	< 0.0001
MNA-1 (ng/mL)	9 (8–15)	13 (8–21)	-2.1	NS
REE (Kcal/day)	1,510 ± 278	1745 ± 364	-3.5	< 0.001

The values are expressed as the means ± SD or medians and interquartile range. The differences between lean and obese subjects were evaluated using Student’s t-test or Mann–Whitney U test. SBP = systolic blood pressure, DBP = diastolic blood pressure, BMI = body mass index, HDL = high-density lipoproteins, LDL = low-density lipoproteins, HOMA-IR = homeostasis model assessment insulin resistance, MNA-1 = *N*^*1*^-methylnicotinamide, REE = resting energy expenditure. P values adjusted for the effects of age.

For the polymorphism rs694539 *NNMT*, the AA genotype frequency was significantly higher in lean subjects, but no differences in allelic frequencies were found (Table 2). For the rs1941404 *NNMT*, rs1421085 *FTO* and rs3751723 *IRX3* polymorphisms, no differences in allelic and genotypic frequencies were found between lean and obese groups. The genotype frequencies of polymorphisms were consistent with the Hardy–Weinberg proportions, with exception of the rs1421085 (Table 2).

**Table 2:** Distribution of allelic and genotype frequencies of the SNPs for each group.

	Lean subjects n = 100	Obese subjects n = 100	X^2^y	*p*
*rs694539 NNMT Genotypes*				
GG	57 (0.57)	66 (0.66)	X^2^y = 6.78	< 0.05
AG	37 (0.37)	34 (0.34)		
AA	6 (0.06)	0 (0)		
*Allelic frequencies*				
G	0.75	0.84	X^2^y = 1.96	NS
A	0.25	0.16		
*rs1941404 NNMT genotypes*	N = 99	N = 97		
TT	42 (0.424)	38 (0.392)	X^2^y = 2.41	NS
TC	48 (0.485)	43 (0.443)		
CC	9 (0.091)	16 (0.165)		
*Allelic frequencies*				
T	0.67	0.61	X^2^y = 0.54	NS
C	0.33	0.39		
*rs1421085 FTO genotypes*	N			
TT	56 (0.56)	43 (0.43)	**X**^**2**^** = **4.5	NS
TC	42 (0.42)	52 (0.52)		
CC	2 (0.02)	5 (0.05)		
*Allelic frequencies*				
T	0.77	0.69	**X**^**2**^** = **1.24	NS
C	0.23	0.31		
*rs3751723 IRX3 genotypes*				
GG	43 (0.435)	39(0.40)	**X**^**2**^** = **0.36	NS
GT	45(0.454)	46(0.47)		
TT	11(0.111)	13(0.13)		
*Allelic frequencies*				
G	0.66	0.34	**X**^**2**^** = **0.09	NS
T	0.63	0.37		

() = Percentage.

No differences were found in comparisons of anthropometric and metabolic characteristics between genotypes of the rs694539 polymorphism under the dominant model. However, under the recessive model, weight, BMI, and REE were significantly lower carriers of the AA genotype than were the GG and AG genotypes (Table 3). Similarly, no differences were found in the comparison of anthropometric and metabolic characteristics by genotypes of the rs1941404 polymorphism of the *NNMT* gene, but under the recessive model, the carriers of GG genotype showed marginal differences in BMI (p = 0.06), serum leptin (p = 0.05), and MNA-1 (p = 0.06) concentration (Table 4).

**Table 3:** Anthropometric and metabolic characteristics of rs694539 *NNMT* genotypes (recessive model).

	GG/GA n = 194	AA n = 6	t/z	*P*
**Weight (kg)**	**77 ± 18**	**59 ± 9**	**2.24**	**0.026**
**BMI (kg/m**^**2**^**)**	**28.7 ± 7**	**22.4 ± 1.8**	**2.20**	**0.028**
Glucose (mg/dL)	89 ± 12	82 ± 11	1.26	NS
Total cholesterol (mg/dL)	180 ± 35	169 ± 32	0.73	NS
HDL-c (mg/dL)	51 ± 15	52 ± 12	0.11	NS
LDL-c (mg/dL)	101 ± 36	98 ± 33	1.08	NS
Triglycerides (mg/dL)	109 (77–154)	80 (63–102)	1.22	NS
Insulin (µIU/mL)	10.3 (7.8–15.2)	8.4 (6.4–12)	1.28	NS
Acylated ghrelin (pg/mL)	105 (61–144)	115 (59–121)	0.001	NS
Leptin (ng/mL)	30 (14–50)	24 (10–40)	0.58	NS
HOMA-IR	2.2 (1.6–3.3)	1.7 (1.0–2.5)	1.38	NS
MNA-1 (ng/mL)	11 (7.9 – 18.9)	5.95 (3.0–12.6)	1.62	NS
**REE (Kcal/day)**	**1637 ± 341**	**1,329 ± 312**	**2.18**	** 0.03**

The values are expressed as the means ± SD or medians and interquartile range. The differences between lean and obese subjects were evaluated using Student’s t-test or Mann–Whitney U test. BMI = body Mass Index, HDL = high-density lipoproteins, LDL = low-density lipoproteins, HOMA-IR = homeostasis model assessment insulin resistance, MNA-1 = *N*^*1*^-methylnicotinamide, REE = resting energy expenditure.

**Table 4:** Anthropometric and metabolic characteristics of rs1941404 *NNMT* genotypes (recessive model).

	TT/CT n = 171	CC n = 25	t/z	*P*
Weight (kg)	75 ± 19	79 ± 18	-0.91	NS
BMI (kg/m^2^)	28 ± 7	30.8 ± 7	-1.85	0.06
Glucose (mg/dL)	88 ± 11	89 ± 14	-0.24	NS
Total cholesterol (mg/dL)	179 ± 34	179 ± 39	-0.01	NS
HDL-c (mg/dL)	52 ± 15	50 ± 14	0.66	NS
LDL-c (mg/dL)	102 ± 30	97 ± 46	0.69	NS
Triglycerides (mg/dL)	109 (76–156)	97 (78–128)	1.01	NS
Insulin (µIU/ml)	10.3 (7.3 – 15)	10.3 (7.5–13)	0.015	NS
Acylated ghrelin (pg/mL)	107 (61–147)	90 (61–136)	-0.55	NS
Leptin (ng/mL)	25.6 (13–48)	40.9 (15–63)	-1.9	0.05
HOMA-IR	2.2 (1.6–3.2)	2.3 (1.7–3.3)	0.23	NS
MNA-1 (ng/mL)	9.4 (7.8–14.9)	13.3 (7.8–20.8)	-1.18	NS
REE (Kcal/day)	1628 ± 351	1596 ± 298	0.43	NS

The values are expressed as the means ± SD or medians and interquartile range. The differences between lean and obese subjects were evaluated using Student’s t-test or Mann–Whitney U test. BMI = body Mass Index, HDL = high-density lipoproteins, LDL = low-density lipoproteins, HOMA-IR = homeostasis model assessment insulin resistance, MNA-1 = *N*^*1*^-methylnicotinamide, REE = resting energy expenditure.

Subjects with TC or CC genotypes of rs1421085 *FTO* showed 6 kg and 2 BMI units more than did subjects with the TT wild type, and the circulating MNA-1 concentrations were significantly higher in the subjects with these genotypes (Table 5). For the rs3751723 *IRX3* polymorphism, leptin and MNA-1 concentrations were marginally elevated in subjects with GT/TT genotypes (29 (8.9–41) *vs.* 37.4 (16–52), *p* = 0.05, and 9.9 (7.6–15) *vs.* 14.9 (8–20), *p* = 0.07; respectively).

**Table 5:** Anthropometric and metabolic characteristics of rs1421085 *FTO* genotypes (dominant model).

	TT n = 98	TC/CC n = 102	t/z	*p*
**Weight (years)**	**73 ± 17**	**79 ± 20**	**-2.08**	** 0.038**
**BMI (kg/m**^**2**^**)**	**27.5 ± 6**	**29.5 ± 7**	**-2.13**	** 0.034**
Glucose (mg/dL)	88 ± 11	89 ± 12	-1.1	NS
Total cholesterol (mg/dL)	179 ± 37	180 ± 32	-0.16	NS
HDL-c (mg/dL)	51.4 ± 15	51.2 ± 14	0.06	NS
LDL-c (mg/dL)	101 ± 40	102 ± 31	-0.26	NS
Triglycerides (mg/dL)	102 (71–157)	114 (88–152)	-1.5	NS
Insulin (µIU/mL)	12 ± 8	13 ± 7	-1.0	NS
Acylated Ghrelin (pg/mL)	105 (61–133)	113 (61–156)	-1.0	NS
Leptin (ng/mL)	26 (12–48)	33 (14–53)	-0.9	NS
HOMA-IR	2.1 (1–3)	2.3 (2–4)	-1.8	NS
**MNA-1 (ng/ml)**	**9 (8–15)**	**14 (8–20)**	**2.8**	**0.004**
REE (Kcal/day)	1597 ± 329	1659 ± 357	-1.2	NS

The values are expressed as the means ± SD or medians and interquartile range. The differences between lean and obese subjects were evaluated using Student’s t-test or Mann–Whitney U test. BMI = body mass index, HDL = high-density lipoproteins, LDL = low-density lipoproteins, HOMA-IR = homeostasis model assessment insulin resistance, MNA-1 = *N*^*1*^-methylnicotinamide, REE = resting energy expenditure.

The association of BMI and REE with metabolic variables and SNPs was evaluated using multiple regression analysis including glucose, HDL-C, triglycerides, MNA-1, acylated ghrelin, leptin, insulin concentrations, HOMA-IR, REE or BMI, and testing each one of the rs694539, rs1941404, rs1421085 and rs3751723 SNPs (Table 6). With this model, REE, leptin, MNA-1concentrations, and age were positively associated with BMI and negatively associated HDL-C and acylated ghrelin concentrations. BMI was independently associated with glucose, triglycerides, insulin, MNA-1 concentrations, HOMA-IR, and rs1421085 *FTO* (Table 6). With this model, and including rs1941404, we found a positive association of BMI with REE, age, and leptin and a marginal association with the MNA-1 concentration and rs1941404 (*p* = 0.066). This last association was first observed in the comparison of BMI for rs1941404 genotypes under the recessive model (*p* = 0.06). No other SNPs were associated with BMI under the dominant model. The REE was also strongly associated with BMI (p < 0.00001), leptin (*p* < 0.001), insulin concentration (*p* < 0.001), and insulin resistance (*p* < 0.001). No association of the rs694539 *NNMT*, rs1941404 *NNMT*, rs1421085 *FTO* or rs3751723 *IRX3* polymorphisms with REE were observed under the dominant model.

**Table 6:** Association of BMI with metabolic variables and SNPs (dominant model).

p p	rs1941404 *NNMT* NS NS	Glucose NS < 0.05	HDL-c 0.05 0.0001	Triglycerides 0.05 < 0.001	Acyl-Ghrelin (pg/mL) < 0.01 < 0.01	Leptin (ng/mL) < 0.0001 < 0.0001	Insulin (µIU/mL) NS < 0.0001	HOMA-IR NS < 0.0001	MNA-1 (pg/mL) < 0.05 < 0.001	REE (Kcal/day) < 0.0001 < 0.0001
p p	rs1421085 *FTO* NS 0.01	Glucose NS < 0.05	HDL-c < 0.05 < 0.001	Triglycerides NS < 0.001	Acyl-Ghrelin (pg/mL) < 0.01 < 0.01	Leptin (ng/mL) < 0.0001 < 0.0001	Insulin (µIU/mL) NS < 0.0001	HOMA-IR NS < 0.0001	MNA-1 (pg/mL) 0.05 < 0.001	REE (Kcal/day) < 0.0001 < 0.0001

Multivariate p in first line; univariate fit p in the second line; BMI = body mass index, HDL = high-density lipoproteins, HOMA-IR = Homeostasis model.

assessment insulin resistance, LDL = low-density lipoproteins, MNA-1 = *N*^*1*^-methylnicotinamide, REE = resting energy expenditure, * = mg/dL. Multiple.

regression analysis adjusted by gender and age. Model including rs1941404 *NNMT* explained 0.53 of the variability. Model including rs1421085 FTO.

explained 0.72 of the variability.

Using the same regression model and including the rs1421085 and rs3751723 haplotype, BMI was negatively associated with HDL-C (*p* < 0.05) and acylated ghrelin (*p* < 0.001) and positively associated with leptin (*p* < 0.00001) and REE (*p* < 0.00001). With the univariate fit, BMI was associated with triglycerides (*p* < 0.001) and insulin (*p* < 0.00001) concentrations, insulin resistance (*p* < 0.00001) and CG rs1421085 and rs3751723 haplotype (p = 0.01) after adjusting for gender and age.

No linkage disequilibrium was found between rs694539 and rs1441404 in the *NNMT* gene or between rs1421085 *FTO* and rs3751723 *IRX3*. For generalized multifactor dimensionality reduction (GMDR), we tested three logistic regression models, two models that included the four SNPs with and without the other predictors (i.e., gender, age, HDL-c, LDL-c, triglycerides, REE, acylated ghrelin, leptin, and HOMA-IR), and a third model with only *FTO* rs1421085 and *IRX3* rs3751723 with the predictors. The models with the other predictors showed better accuracy and gene–gene and gene-environmental interactions (Table 7). Our results showed that the four SNPs and metabolic variables interacted in the obesity phenotype adjusted to a multifactorial model, which is consistent with previous reports^24,25^ (Table 8).

**Table 7:** GMDR analysis of rs694539 and rs1941404 *NNMT*, rs1421085 *FTO*, and rs3751723 *IRX3* for obesity.

Phenotypes	Model	Testing accuracy (%)	CVC	OR (95% CI)	p-value
Obese/non-obese	rs694539, rs1941404, rs1421085, rs3751723	0.674	5/5	4.62 (1.75–12.10)	0.001
Obese/non-obese	rs694539, rs1941404, rs1421085, rs3751723*	0.744	5/5	9.86 (1.42–68.40)	< 0.05

* Predictors: gender, age, HDL-c, LDL-c, triglycerides, REE, acylated ghrelin, leptin, HOMA-IR.

**Table 8:** GMDR analysis of rs1421085 *FTO* and rs3751723 *IRX3* for obesity.

Phenotypes	Model	Testing accuracy (%)	CVC	OR (95% CI)	*P*	
Obese/non-obese	rs9939609 *FTO*; rs17817449 *FTO*; rs3751723 *IRX3*	64.86%	10/10	3.50 (2.10–5.90)	0.0001	Srivastava, A., et al. 2015
Obese/non-obese	rs3751723 *IRX3*; IRX3 x *FTO* rs9939609	55.6% 56.4%	10/10 10/10	1.78 (0.27–11.72) 1.90 (0.28–12.83)	NS NS	Ferreira Todendi, P., et al. 2019
Obese/non-obese	rs1421085 *FTO*; rs1421085 *FTO* x rs3751723 IRX3*	55.36% 60.44%	5/10 10/10	1.99 (1.28–3.11) 2.46 (1.71–3.55)	< 0.01 < 0.001	Our study

Predictors: gender, age, HDL-c, LDL-c, triglycerides, REE, ghrelin, leptin, HOMA-IR.

GMDR = generalized multifactor dimensionality reduction. rs1421085 *FTO* = polymorphism rs1421085 in *FTO* gene*;*

rs3751723 *IRX3* = polymorphism in *IRX3* gene.

## Discussion

The present study analyzed the possible associations of SNPs of the *NNMT* gene and *FTO* and *IRX3* with BMI, REE, and MNA-1 concentrations. The NNMT enzyme regulates adipose tissue energy expenditure via global changes in histone methylation and increased NAD^+^ content^7^. NAD^+^ is a co-substrate for sirtuins, which are a family of NAD^+^-dependent deacetylases^26^. The NNMT-MNA-1 axis plays an important role as a regulator of energy metabolism^7,27,28^, and SNPs in the *NNMT* gene may be associated with obesity and energy expenditure.

REE was significantly higher in obese subjects in our study, but this finding is controversial. A relatively low REE was proposed as a predictor of long-term weight gain in American-Indian populations^29,30^ and Italian populations^31^. The opposite relationship was found in a Nigerian population, where a higher EE was associated with increased body weight over time^32^. However, in the literature, no previous reports evaluated the relationship of REE to obesity in our population.

MNA-1 is an indicator of NNMT activity, and it is strongly associated with obesity and diabetes^9^, and a positive correlation of MNA-1 with BMI was reported^9^. In accordance with these data, we found significantly higher circulating MNA-1 concentrations in obese subjects and a positive correlation with BMI. T2DM patients showed an approximately two-fold increased expression of *NNMT* in WAT compared to that in control subjects and elevated circulating MNA-1 in serum^9^ and urine^10^. However, information on serum MNA-1 in obesity is scarce.

Previous reports described that an A allele and mutated AA genotype of the rs694539 *NNMT* gene were associated with non-alcoholic steatohepatitis and the stage of non-alcoholic fatty liver disease^11^. We analyzed this polymorphism in healthy subjects under the recessive model and found that weight, BMI, and REE were significantly lower in the AA genotype than in the other genotypes. The rs694539 polymorphism of the *NNMT* gene is located in a noncoding region that affects the regulation of transcription^33^. This location suggests that genetic variants affect the expression of NNMT. The *NNMT* gene is expressed at high levels in adipose tissue^8^ in obesity and diabetes^10^. We interpret our results with caution due to the limited information in the literature on this subject. To our knowledge, this study reports the first finding and requires confirmation.

In accordance with our results, the rs1941404 genotype effect was reported as recessive. Zhu et al.^15^ found an association of the rs1941404 CC genotype with hyperlipidemia and lower REE, which is an important factor leading to obesity. In our recessive model, individuals with the CC genotype showed marginally higher BMI, leptin, and MNA-1 concentrations. These differences may result from the inclusion of subjects with higher BMI than the subjects included by Zhu et al.^15^. The trend of higher MNA-1 concentrations in carriers of the rs1941404 CC genotype was not reported. Additional work is needed to confirm this finding.

Some variants within the first intron of the *FTO* gene, such as rs9939609, rs8050136, rs9930506, rs17817449, and rs1421085, are in strong linkage disequilibrium and are associated with increased body weight, body fat, BMI, waist circumference, and energy intake^34,35^. The BMI-increasing allele of the rs1421085 *FTO* was associated with higher protein intake^36^, more eating episodes per day, and total energy intake^37^. Subjects with the risk C allele of rs1421085 FTO were characterized with a higher perception of hunger, which may result in higher food intake^21^. The rs1421085 was associated with obesity risk in North India^25^, which is consistent with our data of higher weight and BMI in subjects with the TC/CC genotypes. In accordance with this report, no linkage disequilibrium was found between rs1421085 *FTO* and rs3751723 *IRX3*^25^.

The obesity-associated SNPs in the first intron of *FTO* were functionally connected to *IRX3*. These SNPs directly interact with the promoters of *FTO* and *IRX3* in human and mouse genomes^22^. The *FTO* gene regulates *IRX3* and *IRX5* genes expression, which play a role in white and brown adipose tissue differentiation during the fetal period. The low expression level of these genes is associated with the development of less white adipose tissue and higher brown adipose tissue (BAT), and thermogenesis^20^. Therefore, an increased *IRX3* and *IRX5* expression in rs1421085 risk allele carriers, favors a greater amount of white adipose tissue and a consequently higher BMI. This finding is observed in double CC risk allele carriers of this SNP who have a 1.7-fold higher obesity risk compared to the homozygous TT genotype^20^. In accordance with these data, we found that subjects with one or two risk allele (TC or CC) of rs1421085 *FTO* had 6 kg and 2 BMI units more than did subjects with the TT genotype. Genetic variants of the *FTO* and *IRX3* genes are in high linkage disequilibrium and associated with obesity. We found the TG haplotype of rs1421085 was more frequently associated with obesity in Mexican subjects. Our work did not find an association of these SNPs with REE, but the editing of rs1421085 in adipocytes from a person with the risk allele restored *IRX3* and *IRX5* repression, activated the browning expression program and restored thermogenesis^20^. The rs9939609 *FTO* was associated with BMI in several reports in Mexican populations^38,39^, but rs1421085 *FTO* was reported in only one study. The present study is the first work that has analyzed the associations of BMI and REE with the genetic variant in the *NNMT* gene, similar to the association of the rs1421085 *FTO* and rs3751723 *IRX3* haplotypes. We also reported the interaction of four SNPs and metabolic variables with the obesity phenotype in a Mexican population.

In conclusion, REE and MNA-1 concentrations were significantly higher in obese subjects. BMI was strongly associated with REE, negatively associated with HDL-C, acylated ghrelin, and positively associated with leptin, and MNA-1 concentrations. The univariate fit showed a positive association of BMI with triglycerides, insulin, and HOMA-IR. The REE was strongly associated with age, BMI, insulin, and insulin resistance. Under the recessive model, subjects with the rs694539AA genotype had lower weight, BMI and REE, and subjects with the rs1941404GG genotype showed higher BMI, leptin, and MNA-1 concentrations than subjects with the other genotypes. The rs1421085 *FTO* polymorphism and the rs1421085 *FTO* and rs3751723 *IRX3* haplotypes were associated with BMI but not with REE. Using the GMDR approach, we found that the obesity phenotype adjusted to a multifactorial model, with the interaction of the four SNPs and metabolic variables.

## Material and methods

### Subjects

We recruited 200 clinically healthy subjects 30 to 50 years old: 100 subjects (32%/68% men/women) with a BMI between 18.5–25 kg/m^2^ and 100 subjects (35%/65% men/women) with BMI > 30 kg/m^2^.

### Anthropometric measurements and blood pressure (BP)

Weight was measured using a Roman type Tanita BC533 scale. Height was measured using a SECA 406 Stadiometer to calculate the BMI. Systolic and diastolic blood pressures were measured in a sitting position after a 10-min rest. All measurements were performed in duplicate.

### Resting energy expenditure (REE)

REE was evaluated via indirect calorimetry (IC) using the Fitmate device (Wellness Technology, Cosmed, USA). The instrument was calibrated before each assessment, following the manufacturer’s specifications. For evaluation, the participants were instructed to avoid physical exercise and the consumption of coffee or black tea for 24 h before examination. The measurements were performed in a quiet room with a controlled temperature between 22-24ºC after a 12-h overnight fast, low light and no noise. The participants remained awake and lying in a supine position throughout the evaluation. Oxygen consumption (O_2_ production) was measured for 15 min. The first 10 min was discarded for a better steady state.

### Biochemical measurements

Venous blood samples were taken after an overnight fast for the measurement of serum glucose, lipid profile, insulin, leptin and acylated ghrelin concentrations and DNA extraction. Serum glucose and lipid profile were measured using enzymatic methods with a chemical analyzer (Auto KEM II, Kontrollab, Italy). Insulin, leptin and acylated ghrelin concentrations were measured using an ELISA kit (ALPCO Immunodiagnostic AG, Stubenwald-Allee, Bensheim, USA) with a sensitivity of 0.399 µIU/mL (range 3.0–200 µIU/mL) for insulin, a sensitivity of 0.42 ng/mL (normal range 1–100 ng/mL) for leptin, and 5 pg/mL (range 2.0–250 pg/mL) for acylated ghrelin. Insulin resistance (IR) was calculated using the homeostatic model assessment (HOMA)^40^. Serum samples were subsequently stored in aliquots at − 80 °C for measurements of circulating MNA-1 levels.

The plasma *N*^*1*^-methylnicotinamide (MNA-1) was measured using liquid chromatography coupled to mass spectrometry (LC–MS) using an electrospray ionization–single quadrupole mass spectrometer (Acquity Arc system with QDa Mass Detector; Waters Corporation, Milford, MA, USA). Briefly, samples were deproteinized with trichloroacetic acid (TCA). Chromatographic separation was performed using a reverse-phase XBridge BEH C18 (particle size 2.5 μm) 100 mm × 3.0 mm column (Waters), protected with a C18 (ODS) 4(L) × 3.0(D) mm guard cartridge (Waters) and maintained at 25ºC. All buffers were filtered through a 0.2 μm filter (Phenomenex) under reduced pressure prior to use. Mobile phase consisted of a 10-mM ammonium formate solution tritiated to pH 2.2 by formic acid. The UHPLC operation was performed in isocratic mode with a constant flow rate of the mobile phase at 0.3 mL/min. ESI ionization was performed in the positive ion mode. The mass spectrometer was operated in selected ion recording (SIR). The single ion mode was used with 137.15 m/z for *N*^*1*^–methylnicotinamide. The quantification was performed using the peak area ratio.

### SNP genotyping

Genomic DNA was isolated from whole blood using a commercial kit. The rs694539 genotyping was performed using PCR–RFLP and the following primers: 5′-AAGTGCTGACAGGTGATAGG-3 '(forward) and 5′-CATCTTTTCACTCTCCTTGC-3′ (reverse) at an aligning temperature of 62 °C for 30 s for 35 cycles. The 187 pb PCR product was digested with the NIaIII restriction enzyme at 37ºC overnight^13^. The digestion PCR products were loaded onto a 3% agarose gel at 80 V for 90 min and visualized using ultraviolet light transillumination (ChemiDoc, Bio-Rad). A band of 187 pb corresponded to the G allele, and two bands of 106 pb and 81 pb were observed for the A allele (13). For genotyping of the *FTO* rs1421085 polymorphism, we used the primers 5´-TAGTAGCAGTTCAGGTCCTAAGGCGTG-3´(forward) and 5′-CAGATTAAGGTGATGGGTTG-3′ (reverse) at a concentration of 10 pmol, 50 ng DNA, 0.2 mM dNTP´S, 2.0 mM magnesium chloride (MgCl2), Taq polymerase 2 units and buffer Taq 1 × using the amplification program previously described^21^. A total of 10% of samples were re-analyzed to check the reliability of genotyping, and a 99% matching was obtained.

Genotyping of *IRX3* rs3751723 and *NNMT* rs1941404 was performed using a validated Taqman® allelic discrimination protocol (Applied Biosystems®). For the IRX3 (VIC/FAM) G/T transversion substitution,

CCGAACAGATTGGCGGAGATTCCCG[G/T]GGCTCCGGGCTCTGATTGACATTTC. For the NNMT (VIC/FAM) A/G transition,

AGATGAGATAGGCCCATGTGTGTGC[A/G]TGTTAGTAAATTTGTGTATGTGCAC, using TaqMan™ Genotyping Master Mix enzyme (Thermo Fisher Scientific, Inc., Waltham, MA, USA). The reactions were performed in a CFX96 Touch (Bio-Rad, USA) using a 96-plate thermal cycler, according to the manufacturer's protocols. A total of 10% of samples were re-analyzed to check the reliability of genotyping, and 99% matching was obtained.

### Statistical analysis

Anthropometric and metabolic data are expressed as the means ± SD or medians (25–75 quartiles). The differences between lean and obese subjects, such as the comparison between genotypes under the recessive model, were evaluated using Student’s t-test or Mann–Whitney *U* test. We compared allelic and genotypic frequencies between groups using χ^2^ with Yates correction. Associations of BMI and REE with metabolic, hormonal and SNPs variables were evaluated with a multiple regression analysis, and relationships between variables were analyzed used the Spearman correlation coefficient. Analyses were performed using the statistical package Statistica 7 (Statsoft Inc., Tulsa, OK, USA).

Analyses were performed using SNP & Variation Suite v8.8.1 (Golden Helix, Inc., Bozeman, MT, www.goldenhelix.com). The four DNA variants were analyzed for deviation from the Hardy–Weinberg equilibrium using Fisher's exact test. *P* < 0.01 indicated a statistically significant difference. For the association study, we used a stepwise linear regression model (https://doc.goldenhelix.com/SVS/latest/svsmanual/numeric_regression.html) with recoded genotypes with the additive genetic model (DD = 2, Dd = 1, dd = 0). BMI and REE were used as response variables. Multiple Bonferroni and false discovery rate corrections indicated a statistically significant difference. To analyze possible interactions between *FTO-IRX3* (25, 26), we used the generalized multifactor dimensionality reduction (GMDR) approach [25]. The model that maximized testing accuracy and cross-validation consistency (CVC) was chosen as the best model (version 0.9; https://www.ssg.uab.edu/gmdr/). A *p*-value < 0.05 was considered statistically significant.

## Statements of ethics approval and consent to participate

The Institutional Ethics Committee of the University of Guanajuato (ClBIUG-P-12–2015) approved this study. All participants signed written informed consent to participate in the study. The study was performed in accordance with the ethical standards of the Declaration of Helsinki in 1983 and in agreement with the Good Clinical Practice guidelines.

## Supplementary information

Supplementary file1 (XLSX 42 kb)

## Data Availability

The data are available to request and under the safeguard of Dr. Elva Perez-Luque and Dr. Nicté Figueroa-Vega. (https://www.dropbox.com/home/PROJECTS/Gasto%20calórico/SNP_NNMT).
